# Carbonic Anhydrase Inhibitors Part 72^1^ Synthesis and Antiglaucoma Properties of Metal Complexes of *p*-Fluorobenzolamide

**DOI:** 10.1155/MBD.1999.67

**Published:** 1999

**Authors:** Claudiu T. Supuran, Andrea Scozzafava, Luca Menabuoni, Francesco Mincione, Fabrizio Briganti, Giovanna Mincione

**Affiliations:** 1 Università degli Studi Dipartimento di Chimica Laboratorio di Chimica Inorganica e Bioinorganica Via Gino Capponi 7 Firenze I-50121 Italy; 2 Ospedale Santa Maria Nuova U. O. Oculistica Via della Pergola 60 Firenze I-50121 Italy; 3 Università degli Studi Istituto Oculistico Viale Morgagni 85 Firenze I-50134 Italy

## Abstract

Metal complexes of a heterocyclic sulfonamides possessing very strong carbonic anhydrase (CA) inhibitory properties, i.e., 5-(*p*-fluorobenzenesulfonylamido)-1,3,4-thiadiazole-2-sulfonamide (p-fluorobenzolamide) were prepared. The new complexes contained metal ions such as Zn(II), Cu(II), Co(II), Ni(II), Cd(II) and Mn(II). The new compounds were characterized by standard physico-chemical procedures, and assayed as inhibitors of three CA isozymes, CA I, II and IV. Very good inhibition has been evidenced both for the parent sulfonamides as well as for the prepared complexes, against all three investigated isozymes. Some of these new complexes as well as the parent sulfonamide, strongly lowered intraocular pressure (IOP) in normotensive rabbits when administered as a 2% solution into the eye.


### CARBONIC ANHYDRASE INHIBITORS. Part 72[1] SYNTHESIS AND ANTIGLAUCOMA PROPERTIES OF METAL COMPLEXES OF *p*-FLUOROBENZOLAMIDE

Claudiu T. Supuran[1]*, Andrea Scozzafava,[1] Luca Menabuoni[2], Francesco Mincione[3], Fabrizio Briganti[1] and Giovanna Mincione[1]

[1] Università degli Studi, Dipartimento di Chimica, Laboratorio di Chimica Inorganica e Bioinorganica, Via Gino Capponi 7, I-50121, Firenze, Italy

[2] Ospedale Santa Maria Nuova, U. O. Oculistica, Via della Pergola 60, I-50121, Firenze, Italy

[3] Università degli Studi, Istituto Oculistico, Viale Morgagni 85, I-50134 Firenze, Italy

**Abstract**: Metal complexes of a heterocyclic sulfonamides possessing very strong carbonic anhydrase (CA) inhibitory properties, i.e., 5-(*p*-fluorobenzenesulfonylamido)-1,3,4-thiadiazole-2-sulfonamide (p-fluorobenzolamide) were prepared. The new complexes contained metal ions such as Zn(II), Cu(II), Co(II), Ni(II), Cd(II) and Mn(II). The new compounds were characterized by standard physico-chemical procedures, and assayed as inhibitors of three CA isozymes, CA I, II and IV. Very good inhibition has been evidenced both for the parent sulfonamides as well as for the prepared complexes, against all three investigated isozymes. Some of these new complexes as well as the parent sulfonamide, strongly lowered intraocular pressure (IOP) in normotensive rabbits when administered as a 2% solution into the eye.

**Introduction**

The sulfonamides represent an important class of biologically active compounds, with at least five different classes of pharmacological agents that have been obtained from the sulfanilamide structure as lead, the derivative initially studied by Domagk [2] as the first modern chemotherapeutic drug. Indeed, the antibacterial sulfonamides [3] continue to play an important role in chemotherapy, alone or in combination with other drugs [4], the sulfonamides that inhibit the zinc enzyme carbonic anhydrase (CA, EC 4.2.1.1) possess many applications as diuretic, antiglaucoma or antiepileptic drugs among others [5-7], the hypoglycemic sulfonamides are extensively used in the treatment of some forms of diabetes [8], whereas the thiazides and high-ceiling diuretics might be considered as a fortunate development of the CA inhibitors [9], but these compounds possess a different pharmacological profile, independent of CA inhibition [10,11]. Finally, some antithyroid drugs have also been developed starting from the sulfonamide structure as lead molecule [12].

The second class of the above mentioned pharmacological agents, i.e., the sulfonamides with CA inhibitory action, have thoroughly been investigated in the last 10 years, mainly in the search of a topically effective antiglaucoma drug [13-19]. The possibility of administering a sulfonamide via the topical route directly into the eye, although investigated in the 1950-s [20,21], has been totally unsuccessful, whereas the systemic administration, quite useful in lowering intraocular pressure (IOP), was generally accompanied by undesired side effects, due to CA inhibition in other tissues than the eye [21]. In 1983, Maren's group [13] postulated that a water-soluble sulfonamide, also possessing a relatively balanced lipid solubility, would be an effective IOP lowering drug via the topical route, but at that moment no inhibitors possessing such physico-chemical properties existed. They started to be developed in several laboratories soon thereafter [13-19], and in 1995 the first such pharmacological agent, dorzolamide **1** entered in clinical use in USA and Europe [22]. A second compound, brinzolamide **2**, quite similar structurally with dorzolamide has also recently been approved for the topical treatment of glaucoma in USA [23].

In addition to the above-mentioned sulfonamides, we developed in our laboratory the synthesis and characterization of CA inhibitors based on the classical ring systems containing the 1,3,4-thiadiazole-2-sulfonamide moiety [24-26]. Thus, some derivatives structurally related to benzolamide **3**, such as its *p*-fluoro- congener **4**, proved to act as very strong *in vitro* CA inhibitors, possessing at the same time a larger water solubility as compared to the classical clinically used inhibitors, such as acetazolamide **5** for instance [27]. Since we have recently shown that metal complexes of aromatic/heterocyclic sulfonamides possess strong antiglaucoma action in normotensive and glaucomatous rabbits [28,29], it appeared of interest to obtain metal complexes of a benzolamide-like compound, and the *p*-fluoro-derivative **4** proved to be an interesting candidate for such a study.

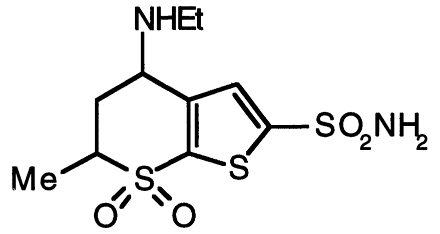

**1**

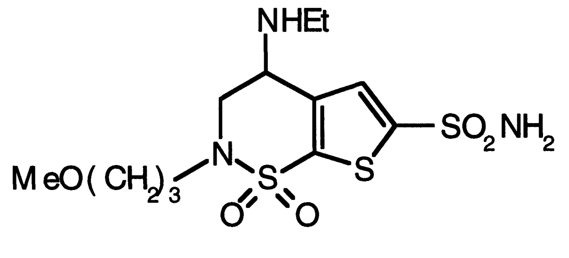

**2**

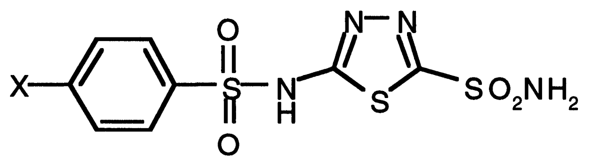

**3**: X = H
**4**: X = F

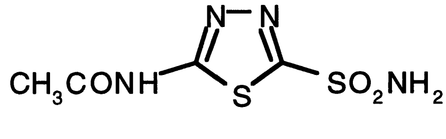

**5**

In this paper we report the synthesis of metal complexes containing the conjugate base of sulfonamide **4** as ligand, and divalent metal ions such as Zn(II), Cu(II), Co(II), Ni(II), Cd(II) and Mn(II). The new compounds reported here were characterized by standard physico-chemical procedures and assayed for inhibition against three physiologically relevant CA isozymes, hCA I, hCA II and bCA IV (h = human, b = bovine isozyme). Good inhibition has been observed with all the new complexes, and especially with the Zn(II) and Cu(II) derivatives. *In vivo* data in normotensive rabbits proved that both the parent sulfonamide as well as its Zn(II) and Cu(II) complexes behave as strong antiglaucoma agents, with a potency comparable to that of dorzolamide, the clinically used drug.

**Materials and Methods**

Melting points were recorded with a heating plate microscope and are not corrected. IR spectra were recorded in KBr pellets with a Carl Zeiss IR-80 instrument. $^1$H-NMR spectra were recorded in DMSO-$d_6$ as solvent, with a Bruker CPX200 instrument. Chemical shifts are reported as values, relative to $Me_4Si$ as internal standard. Conductimetric measurements were done at room temperature (1 mM concentration of complex) in DMSO solution with a Fisher conductimeter. Elemental analyses were done by combustion for C, H, N with an automated Carlo Erba analyzer, and gravimetrically for the metal ions, and were 0.4% of the theoretical values. Thermogravimetric measurements were done in air, at a heating rate of 10°C/min., with a Perkin Elmer 3600 thermobalance.

Sulfonamides used as standards in the enzymatic assay (except for **1**), acetazolamide, 4-fluorobenzenesulfonyl chloride used for the preparation of compound **4**, solvents as well as inorganic reagents were from Sigma, E. Merck and Aldrich. 5-Amino-1,3,4-thiadiazole-2-sulfonamide was prepared from acetazolamide by literature procedures [27], by desacetylation with concentrated hydrochloric acid, followed by neutralization with sodium bicarbonate of the corresponding hydrochloride. Dorzolamide hydrochloride **1** was from Merck, Sharp and Dohme or was prepared as described by Ponticello et al [15].

Human CA I and CA II cDNAs were expressed in *Escherichia coli* strain BL21 (DE3) from the plasmids pACA/hCA I and pACA/hCA II described by Forsman et al. [30] (the two plasmids were a gift from Prof. Sven Lindskog, Umea University, Sweden). Cell growth conditions were those described by Lindskog's group [31], and enzymes were purified by affinity chromatography according to the method of Khalifah et al [32]. Enzyme concentrations were determined spectrophotometrically at 280 nm, utilizing a molar absorptivity of 49 $mM^{-1}.cm^{-1}$ for hCA I and 54 $mM^{-1}.cm^{-1}$ for hCA II, respectively, based on $M_r$ = 28.85 kDa for hCA I, and 29.3 kDa for hCA II, respectively [33,34]. bCA IV was isolated from bovine lung microsomes as described by Maren et al, and its concentration has been determined by titration with ethoxzolamide [35].

*Synthesis of 5-(p-fluorobenzenesulfonamido)-1,3,4-thiadiazole-2-sulfonamide* **4**

An amount of 1.80 g (10 mmol) of 5-amino-1,3,4-thiadiazole-2-sulfonamide was suspended in 20 mL of acetone and 50 mL of water. The mixture was magnetically stirred at 4 °C for 10 minutes, then 10.5 mmol of 4-fluorobenzenesulfonyl chloride, dissolved in 10 mL acetone, and a solution obtained from 0.40 g of

NaOH in 10 mL of water were added dropwise for 30 min, and stirring was continued for other 2 hours at 4 °C. After evaporation of the acetone and neutralization of the reaction mixture with 5 % HCl solution, the precipitated crystals were filtered and recrystallized from ethanol. Yield of 68%, as white crystals, m.p. 193-195 ° lit [27] m.p. 192-194°C (from ethanol-water 1:1, v/v); IR (KBr), $cm^{-1}$ :684, 715, 883, 1132, 1170, 1284, 1357, 1380, 1425, 1572, 1604, 3065; $^{1}$H-NMR (DMSO-$d_6$), (δ, ppm): AA'BB',4H,ArH:, δ(A) 7.21, δ(B) 7.40 ppm, J = 7.8 Hz); 8.03 (br s,2H, $SO_2NH_2$); 8.22 (s,1H, $SO_2NH$); $^{13}$C-NMR (DMSO-$d_6$), (δ, ppm: 118.1; 118.7; 130.0; 130.9; 160.1 (C-2 from thiadiazole); 173.1 (C-5 from thiadiazole). Anal., found: C, 28.63; H, 2.00; N, 16.39 %; $C_8H_7FN_4O_4S_3$ requires: C, 28.40; H, 2.09; N, 16.56 %.

*General procedure for the preparation of complexes* **6-11**

An amount of 6 mmol of sodium salt of sulfonamide **4** was prepared by reacting the corresponding sulfonamide with the required amount of an alcoholic 1N NaOH solution, in ethanol as solvent. To this solution was added the aqueous metal salt solution (Zn(II), Cu(II), Co(II), Cd(II), Mn(II) chlorides, and Ni(II) sulfate), working in molar ratios $RSO_2NH^-$ : $M^{n+}$ of 2:1. The aqueous-alcoholic reaction mixture was heated on a steam bath for one hour, adjusting the pH at 7 if necessary, and after being cooled at 0 °C the precipitated complexes were filtered and thoroughly washed with alcohol-water 1:1 (v/v) and air dried. Yields were in the range of 85-90 %. The obtained powders of compounds **6-11** melted with decomposition at temperatures higher than 350 °C, and were poorly soluble in water and alcohol, but had good solubilities in DMSO, DMF as well as mixtures of DMSO-water, DMF-water.

*Pharmacology*

*Carbonic anhydrase inhibition*

Initial rates of 4-nitrophenyl acetate hydrolysis catalysed by different CA isozymes were monitored spectrophotometrically, at 400 nm, with a Cary 3 instrument interfaced with an IBM compatible PC [36]. Solutions of substrate were prepared in anhydrous acetonitrile; the substrate concentrations varied between $2.10^{-2}$ and $1.10^{-6}$ M, working at 25°C. A molar absorption coefficient ε of 18,400 $M^{-1}.cm^{-1}$ was used for the 4-nitrophenolate formed by hydrolysis, in the conditions of the experiments (pH 7.40), as reported in the literature [36]. Non-enzymatic hydrolysis rates were always subtracted from the observed rates. Duplicate experiments were done for each inhibitor concentration, and the values reported throughout the paper are the mean of such results. Stock solutions of inhibitor (1 mM) were prepared in distilled-deionized water with 10-20% (v/v) DMSO (which is not inhibitory at these concentrations [5]) and dilutions up to 0.01 nM were done thereafter with distilled-deionized water. Inhibitor and enzyme solutions were preincubated together for 10 min at room temperature prior to assay, in order to allow for the formation of the E-I complex. The inhibition constant $K_I$ was determined as described by Pocker and Stone [36]. Enzyme concentrations were 3.3 nM for hCA II, 10 nM for hCA I and 34 nM for bCA IV (this isozyme has a decreased esterase activity [37] and higher concentrations had to be used for the measurements).

*Measurement of tonometric IOP*

Adult male New Zealand albino rabbits weighing 2-3 kg were used in the experiments (three animals were used for each inhibitor studied). The experimental procedures conform to the Association for Research in Vision and Ophthalmology Resolution on the use of animals. The rabbits were kept in individual cages with food and water provided *ad libitum*. The animals were maintained on a 12 h: 12 h light/dark cycle in a temperature controlled room, at 22-26 °C. Solutions of inhibitors (2 %, by weight) were obtained in DMSO-water (2:3, v/v) due to the low water solubility of some of these derivatives. Control experiments with DMSO (at the same concentration as that used for obtaining the inhibitors solutions showed that it does not possess IOP lowering or increasing effects.

IOP was measured using a Digilab 30R pneumatonometer (BioRad, Cambridge, MA, USA) as described by Maren's group [38-40]. The pressure readings were matched with two-point standard pressure measurements at least twice each day using a Digilab Calibration verifier. All IOP measurements were done by the same investigator with the same tonometer. One drop of 0.2 % oxybuprocaine hydrochloride (novesine, Sandoz) diluted 1:1 with saline was instilled in each eye immediately before each set of pressure measurements. IOP was measured three times at each time interval, and the means reported. IOP was measured first immediately before drug administration, then at 30 min after the instillation of the pharmacological agent, and then each 30 minutes for a period of several hours. For all IOP experiments drug was administered to only one eye, leaving the contralateral eye as an untreated control. The ocular hypotensive activity is expressed as the average difference in IOP between the treated and control eye, in this way minimizing the diurnal, seasonal and interindividual variations commonly observed in the rabbit [38-40]. All data are expressed as mean ± SE, using a one-tailed *t* test.

**Results and Discussion**

Reaction of 5-amino-1,3,4-thiadiazole-2-sulfonamide with 4-fluorobenzenesulfonyl chloride in Schotten-Baumann conditions afforded 5-(*p*-fluorobenzenesulfonylamido)-1,3,4-thiadiazole-2-sulfonamide **4**, by the procedure already reported previously by this group [27].

Metal complexes **6-11** containing the conjugate base of sulfonamide **4** (LH) and divalent metal ions, and their elemental analysis data are shown in Table I.

Table I: Prepared complexes **6-11**, containing the conjugate base of sulfonamide **4** (LH) as ligand and their elemental analysis data. L stands for the primary sulfonamide deprotonated species of **4**.

| No. | Complex | Yield (%) | Analysis (calculated/found) %M[a] | %C[b] | %H[b] | %N[b] |
|---|---|---|---|---|---|---|
| **6** | $[MnL_2(OH_2)_2]$ | 75 | 7.1/7.5 | 25.0/25.1 | 2.0/1.7 | 14.6/14.3 |
| **7** | $[NiL_2(OH_2)_2]$ | 65 | 7.6/7.2 | 24.9/24.9 | 2.0/2.3 | 14.5/14.4 |
| **8** | $[CoL_2(OH_2)_2$ | 80 | 7.6/8.0 | 24.9/25.2 | 2.0/2.1 | 14.5/14.1 |
| **9** | $[CuL_2(OH_2)_2]$ | 84 | 8.2/8.1 | 24.8/24.9 | 2.0/2.1 | 14.4/14.3 |
| **10** | $[ZnL_2]$ ] | 87 | 8.8/8.7 | 25.9/25.6 | 1.6/1.3 | 15.1/15.0 |
| **11** | $[CdL_2]$ | 90 | 14.2/14.2 | 24.4/24.5 | 1.5/1.7 | 14.2/14.3 |

[a]By gravimetry; [b]By combustion.

The new complexes have also been characterized by spectroscopic, conductimetric and thermogravimetric measurements (Table II). By comparing the IR spectra of the complexes and the free ligand, the following differences were evidenced: (i) the shift of the two sulfonamido vibrations (both the symmetric as well as the asymmetric one), towards lower wavenumbers in the spectra of the complexes, as compared to the spectra of the corresponding ligand (Table II), as already documented previously for similar complexes [25-29]. One should note that only one pair of such vibrations underwent the above-mentioned shift, presumably those of the primary sulfonamido moiety, whereas the secondary sulfonamide (*p*-F-$C_6H_4SO_2NH$) moiety appeared at the same wavenumbers both in **4** as well as its metal complexes, i.e., at 1132 and 1357 $cm^{-1}$, respectively (data not shown). This is a direct indication that the deprotonated primary sulfonamido moiety of the ligand interacts with the metal ions in the newly prepared coordination compounds; (ii) the C=N stretching vibration in the spectra of the prepared complexes is shifted with 14-21 $cm^{-1}$ towards lower wavenumbers, as compared to the same vibration in the spectrum of sulfonamide **4** indicating that one of the endocyclic nitrogens of the thiadiazolic ring (presumably N-3) acts as donor atom, as already documented by X-ray crystallographic and spectroscopic determinations on complexes of other sulfonamides (such as benzolamide **3**) with divalent metal ions [41] (Table II); (iii) changes in the region 3100-3160 $cm^{-1}$, as the bands present in the spectra of sulfonamide **4** are present in the spectra of complexes **6-11** too, but they are not well resolved, and have a smaller intensity. This is probably due to deprotonation of the $SO_2NH_2$ moiety and participation in the binding of cations (data not shown).

Table II: Spectroscopic, thermogravimetric and conductimetric data for compounds **4-11**.

| **Comp.** | **IR Spectra**[a] $\nu$, $cm^{-1}$ $(SO_2)^s$; $(SO_2)^{as}$ | (C=N) | **Electronic Spectra**[b] $\nu$ ($cm^{-1}$) | **TG analysis**[c] calc./found | **Conductometry**[d] $\Lambda_M$ ($\Omega^{-1}$ x $cm^2$x $mol^{-1}$) |
|---|---|---|---|---|---|
| **4** | 1170; 1380 | 1604 | - | - | 3 |
| **6** | 1150; 1350 | 1583 | 25500; 24200; 19700 | 4.7/5.0[e] | 5 |
| **7** | 1150; 1360 | 1580 | 24100; 17500; 11050 | 4.6/4.5[e] | 6 |
| **8** | 1150; 1355 | 1584 | 26300; 19850; 17100 | 4.6/4.8[e] | 8 |
| **9** | 1155; 1350 | 1590 | 25000; 16900; 14350 | 4.6/4.5[e] | 5 |
| **10** | 1145; 1350 | 1586 | - | f | 4 |
| **11** | 1140; 1300 | 1590 | - | f | 7 |

[a] In KBr; [b]In MgO as standard, by the diffuse reflectance technique; [c]Weight loss between 70-250 °C; [d] 1 mM solution, in DMF, at 25°C; [e]Corresponding to two coordinated water molecules lost at 150-170°C, [f] No weight loss seen under 250 °C.

Diffuse reflectance electronic spectroscopic data (for paramagnetic transition metal ions) helped us to establish the geometry of the cations in the prepared complexes **6-9** (Table II). Thus, octahedral structures were detected for the majority of the prepared complexes (such as the Mn(II); Ni(II); Co(II); and Cu(II) derivatives), whereas tetrahedral geometries were proposed for the Zn(II) and Cd(II) complexes **10** and **11**, based on the stoichiometry and analogy with other structurally related compounds [41-45], which have been characterized by means of X-ray crystallography [41]. TG data confirmed the above-mentioned statements, as two coordinated water molecules were detected in the octahedral complexes **6-9**, whereas conductimetric measurements proved the non-electrolyte nature of all these complexes (Table II).

Thus, similarly to benzolamide **3** [41], the donor system of *p*-flluorobenzolamide **4** probably consists of the sulfonamido nitrogen and endocyclic N-3 atoms when interacting in deprotonated state with the metal ions considered in the present work. Proposed structures for the obtained complexes are shown below.

Table III. CA inhibition data with the standard inhibitors **1-5**, and the metal complexes **6-11** against isozymes CA I, II and IV.

| No | Inhibitor | | $K_I$ (nM) | |
|---|---|---|---|---|
| | | hCA I[a] | hCA II[a] | bCA IV[b] |
| **1** | Dorzolamide | 50000 | 9 | 45 |
| **2** | Brinzolamide | - | 3 | 45 |
| **3** | Benzolamide | 15 | 9 | 12 |
| **4** | *p*-Fluorobenzolamide | 4 | 4 | 7 |
| **5** | Acetazolamide | 900 | 12 | 220 |
| **6** | | 3 | 2 | 5 |
| **7** | | 4 | 3 | 2 |
| **8** | | 3 | 2 | 2 |
| **9** | | 2 | 2 | 4 |
| **10** | | 3 | 1.5 | 3 |
| **11** | | 2 | 2 | 3 |

[a] Human (cloned) isozymes; [b] From bovine muscle; [c] From bovine lung microsomes.

The complexes **6-11** together with the standard CA inhibitors **1-5** were assayed for inhibition against four isozymes, hCA I, hCA II, and bCA IV (Table III). As seen from the above data, p-fluorobenzolamide **4**, the ligand used for preparing metal complexes, is more inhibitory than acetazolamide, benzolamide or dorzolamide, having similar potency with brinzolamide against hCA II, but being a much better inhibitor against bCA IV, as compared to the latter sulfonamide. The metal complexes **6-11** are much more inhibitory than the sulfonamide from which they derive **4**, and than all other simple sulfonamides assayed. They behave similarly to the metal complexes of acetazolamide, methazolamide or dorzolamide previously reported by this group, which were all more inhibitory than the parent sulfonamide from which were prepared [26,28,29].

Particularly strong inhibition was observed for the Cd(II); Zn(II) and Cu(II) complexes, especially against CA II and CA IV, the isozymes critical for aqueous humor formation, but all these metal complexes act as particularly powerful CA inhibitors.

*In vivo* IOP lowering experiments were done in rabbits with the new compounds prepared in the present work, as well as the sulfonamides **1** and **4** for comparison. Some of the IOP lowering data at half an hour and one hour after the instillation of one drop of 2 % solution of inhibitor within the rabbit eye are shown in Table IV, with dorzolamide **1** (at the same concentration) as standard.

As seen from the above data, the sulfonamide **4** is a much more effective IOP lowering agent, as compared to the clinically used compound dorzolamide, at all periods of measurement. From the data of table IV, it is obvious that the metal complexes of sulfonamide **4**, of types **6-11**, investigated by us behave as much more effective IOP lowering agents than dorzolamide or the parent ligand **4**. The most effective cations for the IOP lowering effect were Zn(II), Cu(II) and Cd(II). A remarkable finding consists in the fact that the metal complexes seem to possess a much longer duration of action of the IOP lowering effect, as seen from the measurements done at one and a half hour, which show a reduced pressure (of more than 7 mm Hg), whereas dorzolamide already lost much of its effect. Probably the pharmacokinetic properties of such metal complexes constitute a valuable feature for developing novel types of more effective topical antiglaucoma drugs from this class of compounds.

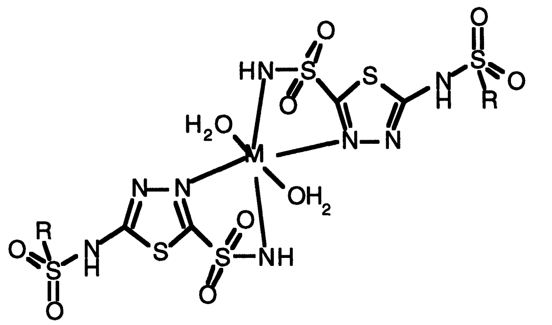

**6-9**

M = Mn(II); Ni(II); Co(II); Cu(II)

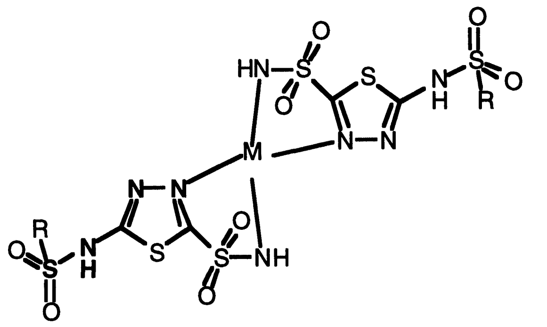

**R = 4-FC$_6$H$_4$**

**10: M = Zn**
**11: M = Cd**

Table IV: IOP lowering following topical application of CA inhibitors, half an hour, one hour and one and a half hours after instillation into the eye of a drop (50 (L) of 2 % solution of inhibitor in DMSO-water (2:3, v/v).

| Inhibitor | ΔIOP ± SE [a] (mm Hg) 1/2 h | 1h | 1.5 h |
|---|---|---|---|
| Dorzolamide **1** | 2.2 ( 0.10 | 4.1 ( 0.15 | 2.7( 0.08 |
| **4** | 5.0 (0.10 | 7.3 ( 0.09 | 6.4( 0.11 |
| **6** | 5.9 (0.10 | 7.8 ( 0.10 | 7.0( 0.06 |
| **7** | 6.0(0.09 | 8.0 ( 0.12 | 7.3( 0.08 |
| **8** | 8.0 (0.14 | 8.5 ( 0.21 | 7.4( 0.07 |
| **9** | 8.0 (0.10 | 8.4 ( 0.09 | 7.7( 0.09 |
| **10** | 9.3(0.09 | 12.5 ( 0.12 | 8.6( 0.12 |
| **11** | 8.0 (0.14 | 8.1 ( 0.21 | 7.7( 0.08 |

[a] $\Delta IOP = IOP_{\text{control eye}} - IOP_{\text{treated eye}}$ (N = 3).

**References**

1 Preceeding part of the series: Supuran, C.T., Scozzafava, A., Menabuoni, L., Mincione, F., Briganti, F., Mincione, G. (1999) *Eur. J. Pharm. Sci.*, in press.
2 Domagk, G. (1935) *Dt. Med. Wocheschr.*, **61**, 250-254.
3 Northey, E.H. (1948) "The Sulfonamides and Allied Compounds", Reinhold, New York, pp. 1-267.
4 Mandell, G.L., Sande, M.A. (1990) "Antimicrobial Agents". In *The Pharmacological Basis of Therapeutics*, 8th Edition, Gilman, A.G., Rall, T.W., Nies, A.S., Taylor P., Eds., Pergamon Press, New York, pp 1047-1064.
5 Supuran C.T. (1994) "Carbonic anhydrase inhibitors", in *Carbonic Anhydrase and Modulation of Physiologic and Pathologic Processes in the Organism*, (Puscas, I., Ed) Helicon, Timisoara, pp. 29-111.
6 Weiner, I.M. (1990) Diuretics and other agents employed in the mobilization of edema fluids. In *The Pharmacological Basis of Therapeutics*, 8th Edition, Gilman, A.G., Rall, T.W., Nies, A.S., Taylor P., Eds., Pergamon Press, New York, pp 713-732.
7 Supuran, C.T., Scozzafava, A., Ilies, M.A., Iorga, B., Cristea, T., Briganti, F., Chiraleu, F., Banciu, M.D. (1998) *Eur. J. Med. Chem.*, **33**, 577-595.
8 Boyd, A.E. (1988) *Diabetes* **37**, 847-850.
9 Beyer, K.H., Baer, J.E. (1961) *Pharmacol. Rev.*, **13**, 517-562.
10 Maren, T.H. (1987) *Drug Dev. Res.*, **10**, 255-276.
11 Supuran, C.T., Conroy, C.W., Maren, T.H. (1996) *Eur.J.Med.Chem.*, **31**, 843-846.
12 Maren, T.H. (1976) *Annu. Rev. Pharmacol. Toxicol.*, **16**, 309-327.
13 Maren, T.H., Jankowska, L., Edelhauser, G.F., Sanyal, G. (1983) *Exp. Eye Res.*, **36**, 457-480.
14 Katritzky, A.R., Caster, K.C., Maren, T.H., Conroy, C.W., Bar-Ilan, A. (1987) *J. Med. Chem.*, **30**, 2058-2062.
15 Ponticello, G.S., Freedman, M.B., Habecker, C.N., Lyle, P.A., Schwam, H., Varga, S.L., Christy, M.E., Randall, W.C., Baldwin, J.J. (1987) *J. Med. Chem.*, **30**, 591-597.

16 Boriack, P.A., Christianson, D.W., Kingery-Wood, J., Whitesides, G.M. (1995) *J. Med. Chem.*, **38**, 2286-2291.
17 Supuran, C.T., Popescu, A., Ilisiu, M., Costandache, A., Banciu, M.D. (1996) *Eur. J. Med. Chem.*, **31**, 439-448.
18 Supuran, C.T., Briganti, F., Scozzafava, A. (1997) *J. Enzyme Inhib.*, **12**, 175-190.
19 Supuran, C.T., Scozzafava, A., Popescu, A., Bobes-Tureac, R., Banciu, A., Creanga, A., Bobes-Tureac, G., Banciu, M.D. (1997) *Eur. J. Med. Chem.*, **32**, 445-452.
20 Becker, B. (1955) *Am. J. Ophthalmol.*, **39**, 177-183.
21 Maren, T.H. (1967) *Physiol. Rev.*, **47**, 595-782.
22 Ponticello, G.S., Sugrue, M.F., Plazonnet, B., Durand-Cavagna, G. (1998) *Pharm. Biotechnol.*, **11**, 555-574.
23 Silver, L.H. (1998) *Am. J. Ophthalmol.*, **126**, 400-408.
24 Supuran, C.T., Clare, B.W. (1995) *Eur.J.Med.Chem.*, **30**, 687-696.
25 Supuran, C.T. and Scozzafava, A. (1997) *J. Enzyme Inhib.*, **12**, 37-51.
26 a) Mincione, G., Scozzafava, A., and Supuran, C.T. (1997) *Metal Based Drugs*, **4**, 27-34; b) Scozzafava, A. and Supuran, C.T. (1997) *Metal Based Drugs*, **4**, 19-26.
27 Supuran, C.T., Ilies, M.A., and Scozzafava, A. (1998) *Eur. J. Med. Chem.*, **33**, 571-582.
28 Supuran, C.T., Mincione, F., Scozzafava, A., Briganti, F., Mincione, G., and Ilies, M.A., (1998) *Eur. J. Med. Chem.*, **33**, 247-254.
29 Supuran, C.T., Scozzafava, A., Saramet, I., Banciu, M.D. (1998) *J. Enzyme Inhib.*, **13**, 177-194.
30 Forsman C, Behravan G, Osterman A, Jonsson BH (1988) *Acta Chem Scand* **B42**, 314-318.
31 Behravan G, Jonasson P, Jonsson BH, Lindskog S (1991) *Eur J Biochem* **198**, 589-592.
32 Khalifah RG, Strader DJ, Bryant SH, Gibson SM (1977) *Biochemistry* **16**, 2241-2247.
33 Nyman PO, Lindskog S (1964) *Biochim Biophys Acta* **85**, 141-151.
34 Henderson LE, Henriksson D, Nyman PO (1976) *J Biol Chem* **251**, 5457-5463.
35 Maren TH, Wynns GC, Wistrand PJ (1993) *Mol Pharmacol* **44**, 901-906.
36 Pocker Y, Stone JT (1967) *Biochemistry* **6**, 668-678.
37 Baird TT, Waheed A, Okuyama T, Sly WS, Fierke CA (1997) *Biochemistry* **36**, 2669-2678.
38 Maren TH, Bar-Ilan A, Conroy CW, Brechue WF (1990) *Exp Eye Res* **50**, 27-36.
39 Maren TH, Brechue WF, Bar-Ilan A (1992) *Exp Eye Res* **55**, 73-79.
40 Brechue WF, Maren TH (1993) *Invest Ophthalmol Vis Sci* **34**, 2581-2587.
41 Alzuet, G., Casanova, J., Borras, J., Garcia-Granda, S., Gutierrez-Rodriguez, A., Supuran, C.T. (1998) *Inorg. Chim. Acta*, **273**, 334-338.
42. Sacconi, L., Mani, F., Bencini, A. (1987) "Nickel", in "Comprehensive Coordination Chemistry", G. Wilkinson, R. Gillard, J. McCleverty Eds., Pergamon Press, Oxford, Vol. 5 pp. 1-347.
43. Banci, L., Bencini, A., Benelli, C., Gatteschi, D., Zanchini, C. (1982) *Struct. Bonding*, **52**, 37-79.
44. Hathaway, B.J. (1987) "Copper", in "Comprehensive Coordination Chemistry", Vol. 5, G. Wilkinson, R.D. Gillard, J. Cleverty Eds., Pergamon, New York, pp. 533-546
45. Lever, A.B.P. (1984) "Inorganic Electronic Spectroscopy", 2nd Edition, Elsevier, Amsterdam, pp. 135-138.

**Received: January 19, 1999 - Accepted: February 4, 1999 - Received in revised camera-ready format: February 8, 1999**

